# Using Clear Aligners to Treat a Surgical Class III Malocclusion: Case Report

**DOI:** 10.1155/crid/3436672

**Published:** 2026-03-08

**Authors:** Feras Y. Dahhas, Kamel M. Alraei, Hashim A. Alzahrani, Sufana A. Alattas, Raghad M. Alharbi

**Affiliations:** ^1^ Alnoor Hospital, Makkah, Saudi Arabia; ^2^ Security Forces Hospital, Makkah, Saudi Arabia, sfh.med.sa; ^3^ National Guard Hospital, Jeddah, Saudi Arabia, ngha.med.sa

## Abstract

**Introduction:**

This case report describes the treatment of a 37‐year‐old male who presented in an orthodontic clinic with a protruded lower jaw and difficulty chewing with his front teeth. The case was treated by a clear aligner with an arch bar and orthognathic surgery. Also, this report describes alternative methods to facilitate maxillomandibular repositioning and stabilization.

**Method:**

The treatment plan includes (1) presurgical alignment and leveling of the teeth by clear aligners (Eon), (2) insertion of the arch bar, (3) mandibular setback surgery, (4) postsurgical fixation using elastics and removal of the arch bar, (5) re‐evaluating the need for refinements or reusing the previous aligners, and (6) retention by using an upper Essix retainer and a lower fixed retainer from the canine to canine overlay by Essix retainer. The patient allowed personal data processing and informed consent was obtained. The informed consent was obtained from the patient.

**Result:**

Correction of skeletal and dental Class III malocclusion was obtained (Class I canine in both sides). Midlines were corrected with positive overjet, and overbite and patient′s profile and facial balance were improved.

**Conclusion:**

This treatment approach successfully met the patient′s demands and resulted in high levels of satisfaction with the treatment outcomes.

## 1. Introduction

A malocclusion has been defined as a significant deviation from the ideal occlusal relationship that may be considered aesthetically or functionally unsatisfactory. The causes of malocclusion can be a complex mix of etiological causes, including hereditary and environmental factors [1]. The prevalence of malocclusions varies depending on the examination. Many factors contribute to this, including the timing of the study, the geographical area, and the criteria used for sample selection, such as age and gender. For example, a study conducted by Alharbi included a group of 680 male subjects with an average age of 12.3 years. According to the results, 84.9% of students had Class I molar relationship [2, 3]. Angle′s Class I malocclusion was the most common type, followed by Classes III and II malocclusion, respectively [4].

Managing Class III malocclusion has always posed challenges for clinicians [5]. Various approaches have been described in the scientific literature, depending on the patient′s age and stage of growth. In prepubertal patients in the early mixed dentition phase, the goal of Class III treatment is to restore normal skeletal growth patterns and improve the occlusal relationship [6]. The most commonly used approaches in growing patients include orthopedic therapy with rapid maxillary expansion and a facemask or other functional appliances [7, 8]. Once the patient reaches the peak of growth, the only viable strategy may be dental compensation without correcting the skeletal malocclusion. Alternatively, if the skeletal discrepancy is significant, it may be necessary to wait until growth is complete and consider orthognathic surgery [9]. Treatment of Class III malocclusion becomes even more complex when there are dental and/or skeletal asymmetries [10].

Many adult patients seek orthognathic surgery later in life, and their concerns and goals may differ from those of younger patients. One common concern is the aesthetic appearance of fixed appliances and the duration of wearing them before and after surgery. Clear aligner orthodontic appliances, consisting of a series of transparent aligners, have gained popularity due to their aesthetic appeal compared to traditional braces [11].

Clear aligner orthodontics has evolved over the past 20 years as an alternative to conventional braces, initially proposed for treating mild malocclusions but gradually expanded to more complex cases [12]. Orthodontists benefit from advantages such as improved financial compensation, reduced office overhead, and increased office efficiency. Patients prefer clear aligners because of their aesthetic benefits, reduced discomfort, fewer orthodontic visits, and the simplicity of changing aligners to align their teeth [13].

Performing orthognathic surgery with clear aligner therapy (CAT) presents a major challenge in achieving intraoperative occlusal control. Although protocols for fixed orthodontic appliances are well established, they are not well documented for CAT cases. Currently, knowledge about CAT with orthognathic surgery is limited, with most information coming from case reports. The adoption of orthognathic surgery for CAT has been cautious and challenging due to factors such as intermaxillary fixation (IMF), postoperative occlusal control, preoperative decompensation management, and long‐term stability, which require further testing and evaluation [12].

In this report, we discuss several alternative methods to facilitate maxillomandibular repositioning and stabilization, including the use of Erich arch bars, Ivy loops, IMF screws, and hybrid arch bar systems. These methods help expand the scope of indications for the clear aligner system and may increase case acceptance among surgical patients seeking esthetic and minimally invasive orthodontic options.

1. Erich arch bars: Erich arch bars are dental appliances used to stabilize the maxilla and mandible in a desired position during orthognathic surgery. They consist of metal bars that are custom‐fitted to the patient′s dental arches and secured with wires or screws. Erich arch bars provide stability and support to the jaws during surgical repositioning.
2. Ivy loops: Ivy loops are small wire loops used in orthodontics and orthognathic surgery to facilitate the movement of teeth and provide anchorage. They are often used in combination with other orthodontic appliances or during the presurgical orthodontic phase to align and position the teeth in preparation for surgery.
3. IMF screws: IMF screws are used to immobilize the maxilla and mandible together during orthognathic surgery. These screws are inserted into specific locations in the jaws and act as temporary fixation points, allowing the surgeon to accurately reposition the jaws and maintain stability during the healing process.
4. Hybrid arch bar systems: Hybrid arch bar systems combine the advantages of traditional arch bars and newer technologies such as mini‐implants or miniplates. They provide stability and control during orthognathic surgery while minimizing the need for IMF. Hybrid arch bar systems offer flexibility and can be customized to meet the specific needs of each patient [14].

These alternative methods help optimize the scope of indications for CAT and may increase the acceptance of surgical patients who are seeking esthetic and minimally invasive orthodontic options. By utilizing these techniques, clinicians can achieve effective maxillomandibular repositioning and stabilization in conjunction with CAT, providing patients with improved outcomes and treatment experiences.

## 2. Case

**Figure 1:**
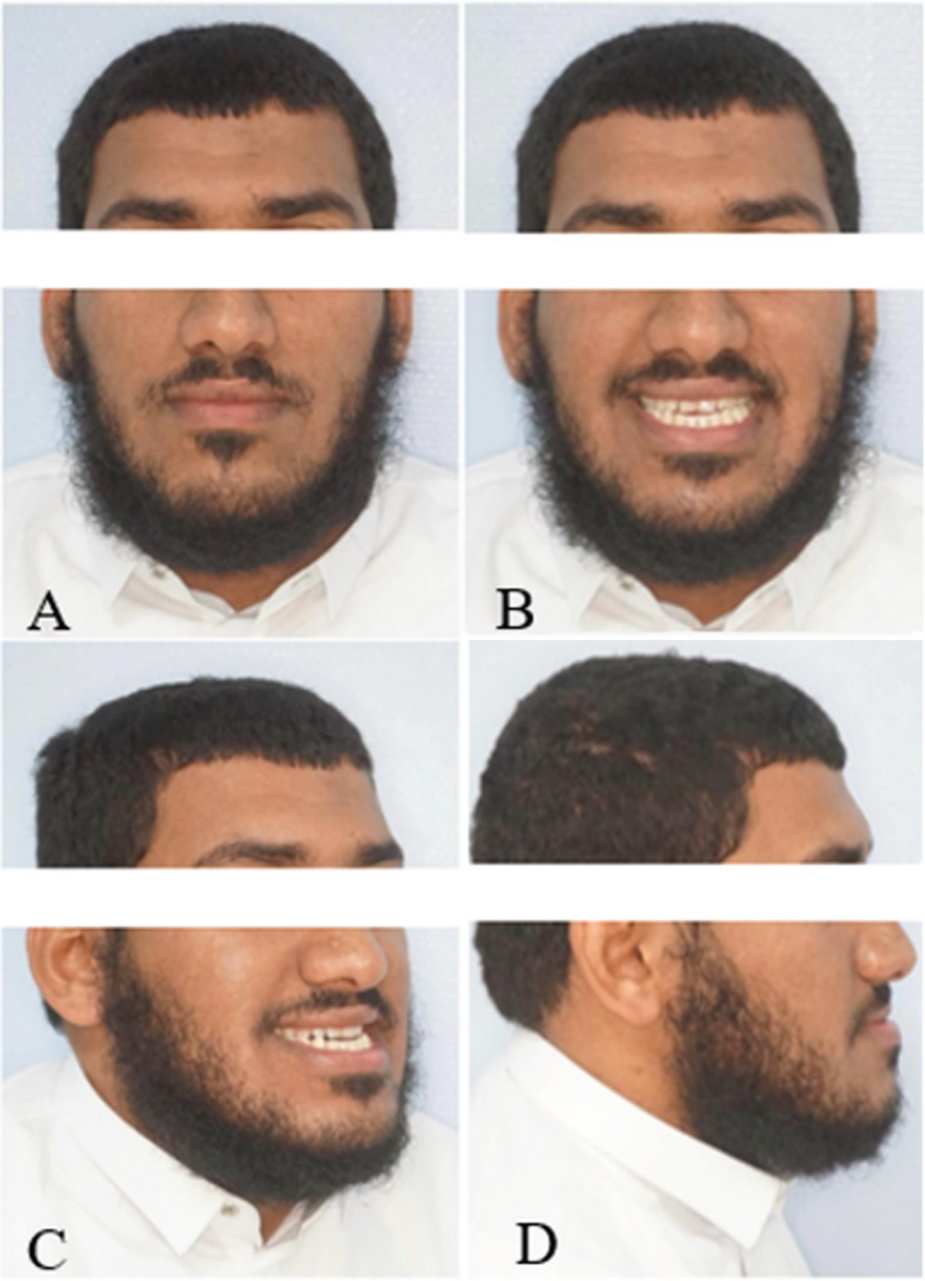
Initial extraoral photos. (A) Mesofacial face type, fairly symmetrical face, increased lower anterior facial height, competent lips, 0‐mm incisal display, average nose size. (B) Eighty‐percent incisal inconsonant smile arc. Upper midline is on the facial midline. Buccal corridors are wider than normal. (C) Slight midface deficiency, no nasal deformity. (D) Concave facial profile. Average nose size. Average nasolabial angle. Average mentolabial sulcus. His chin is protruded and no mentalis strain. Slight increase in mandibular plane. Upper and lower lips are in average length and thickness.

**Figure 2:**
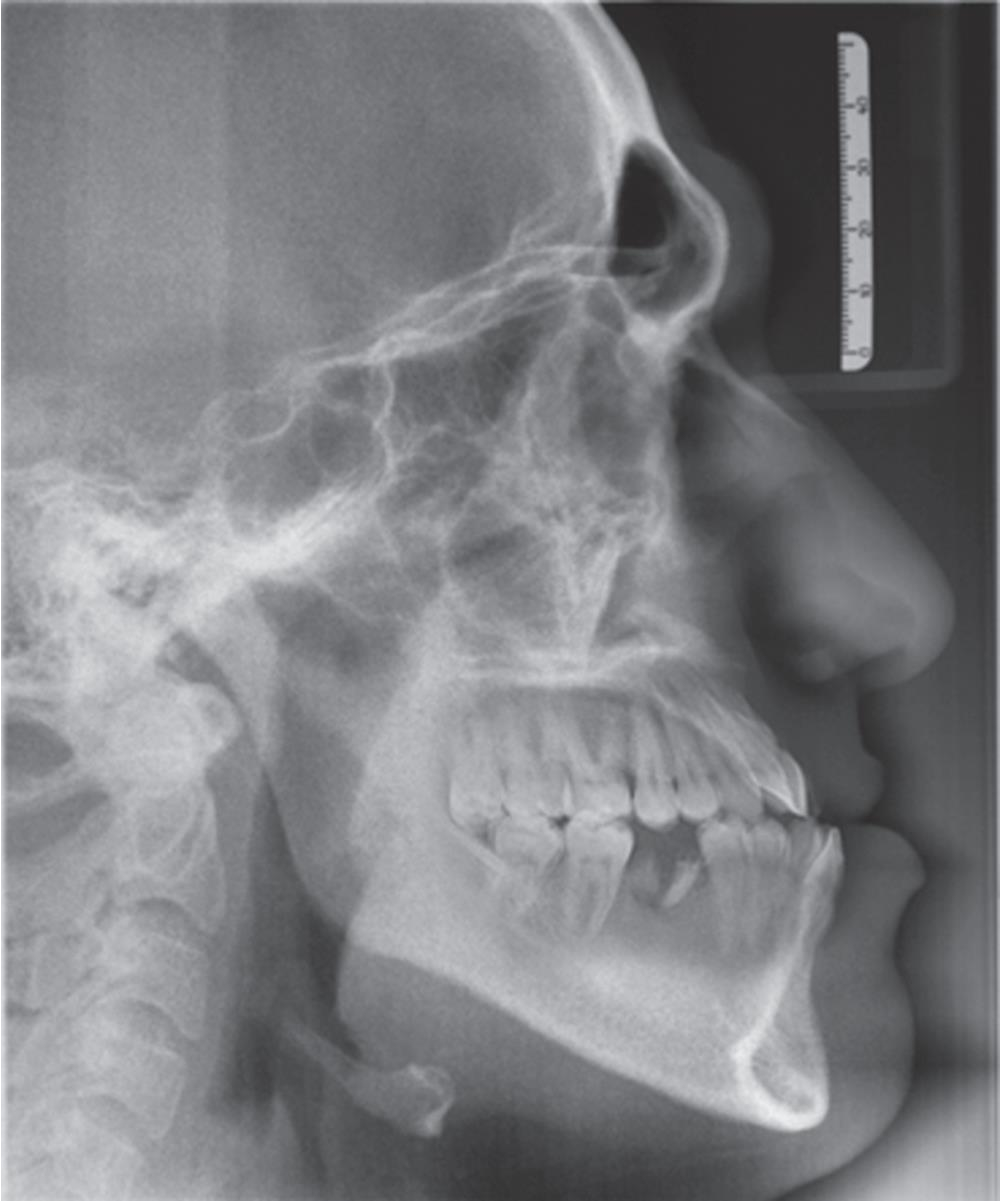
Initial lateral cephalometric radiograph: Class III skeletal pattern according to ANB and Wits, concave skeletal profile, slight increase in mandibular plane, lower anterior face height, upper incisors are proclined and protruded, lower incisors are protruded, upper lip is retruded, lower lip is in average position to esthetic line, and average nasolabial angle and Cervical Stage 6 (CS6).

**Figure 3:**
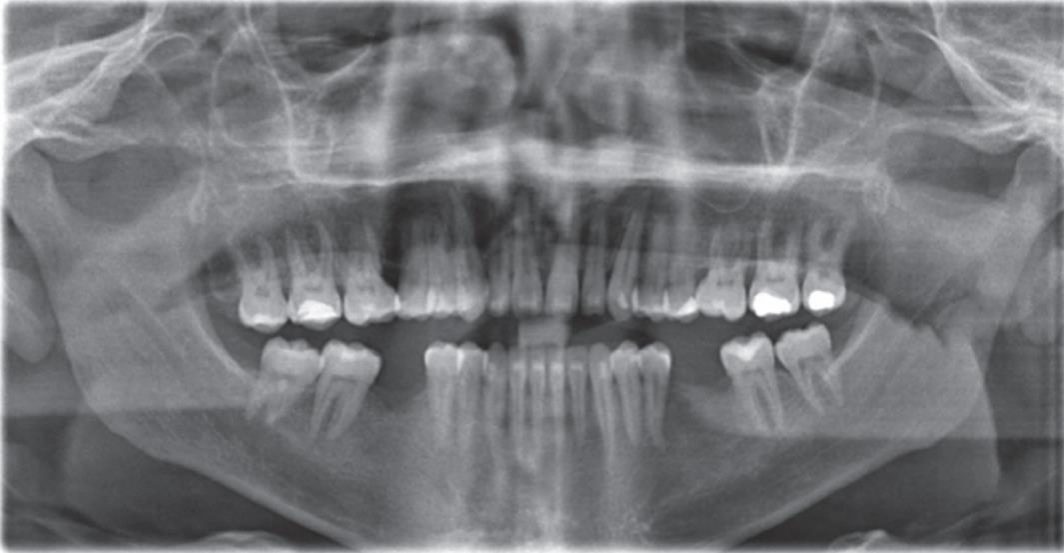
Initial panoramic radiograph: Fairly symmetrical condyles and ramus height, sinuses, and bone trabeculation are within normal limits. All permanent teeth are present except Teeth #36 and 46 (old extractions).

**Figure 4:**
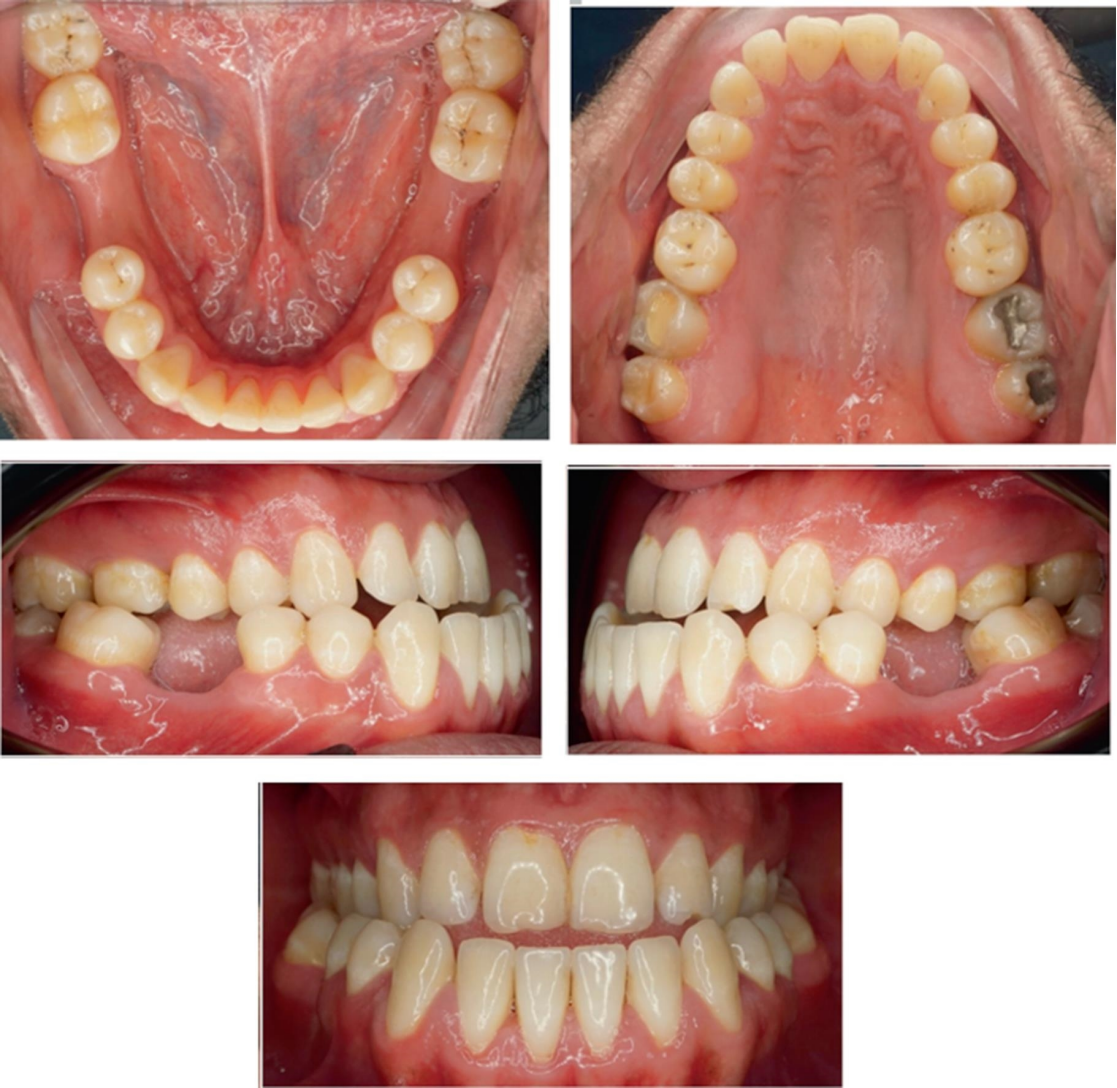
Initial intraoral photos: fair oral hygiene, average tongue size, average frenal attachment, posterior teeth discolored, permanent dentition, average attached gingiva. There is no CR/CO shift; restorations on Teeth #18, 17 and 27; fissure stains on Teeth #16, 26, 38, 47, and 48. Missing Teeth #36 and 46 (old extraction).

**Figure 5:**
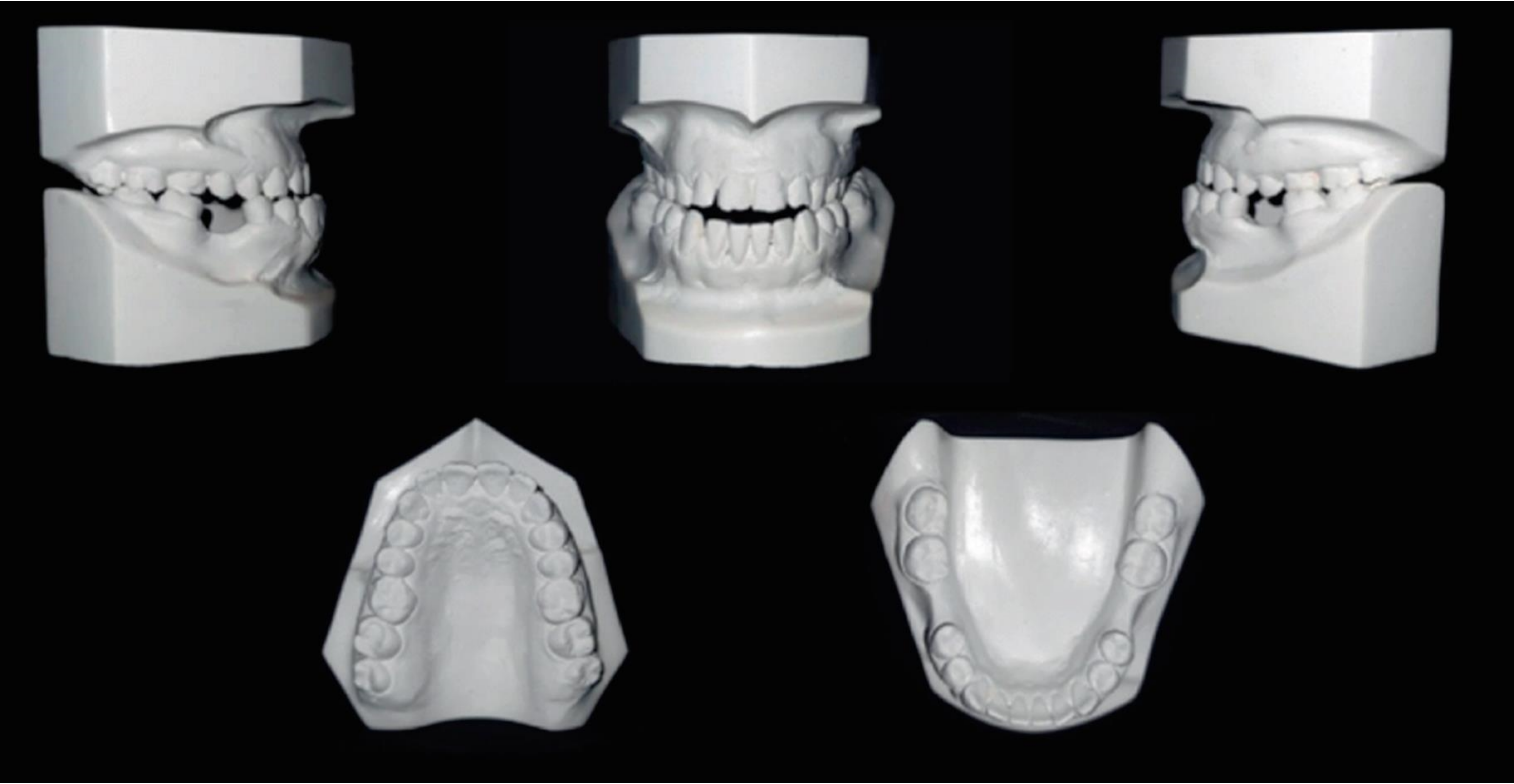
Initial cast photos: permanent dentition, symmetrical upper and lower ovoid arch, canine classification Class III, −5 mm overjet, 4 mm open bite, bilateral posterior true crossbite, lower midline shifted 1 mm to the left, rotated Teeth #16, #11, #12, #24, #32, and #43, intercanine width within normal limits with decrease intermolar width and no Bolton ratio.

This case involves a 37‐year‐old Saudi male who visited the orthodontic clinic with the main concern of having a protruded lower jaw and difficulty chewing with his front teeth. His medical and dental history were unremarkable. The patient had a concave facial profile, with lips that were competent but with the upper lip slightly retruded (Figure 1). Skeletally, he had a Class III skeletal pattern and a slight increase in the vertical dimension (Figures 2 and 3). Dentally, his malocclusion was characterized by a Class III relationship with reversed overjet, anterior open bite, posterior crossbite, a 1‐mm shift of the lower midline to the left, and missing lower first molars (Figures 4 and 5). The cause of the malocclusion was determined to be genetic. The patient′s Index of Orthodontic Treatment Need (IOTN) was Grade 5.m; the patient allowed personal data processing and informed consent was obtained.

The treatment objectives were to correct the skeletal relationship; achieve a Class I canine relationship; correct the midline shift, posterior crossbite, and rotations; and maintain spaces for future prosthetic replacement. The two alternative treatment options considered were comprehensive surgical orthodontic treatment with extraction of the upper fourth premolars and double jaw surgery or comprehensive surgical orthodontic treatment without extraction, specifically mandibular setback surgery.

**Figure 6:**
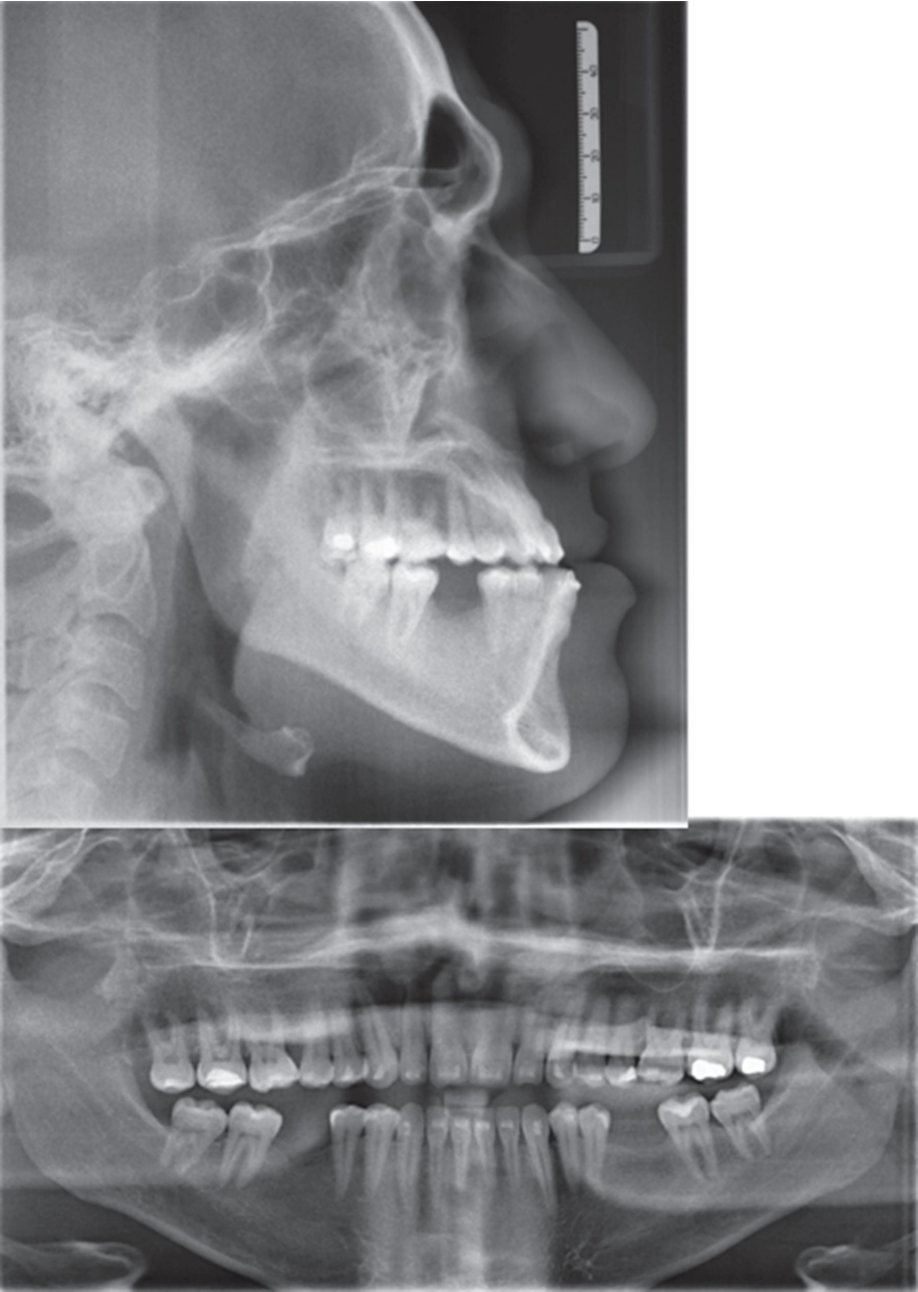
Presurgical lateral cephalometric and panoramic view: after using clear aligners (Eon) to achieve Class l canine relation in both sides.

**Figure 7:**
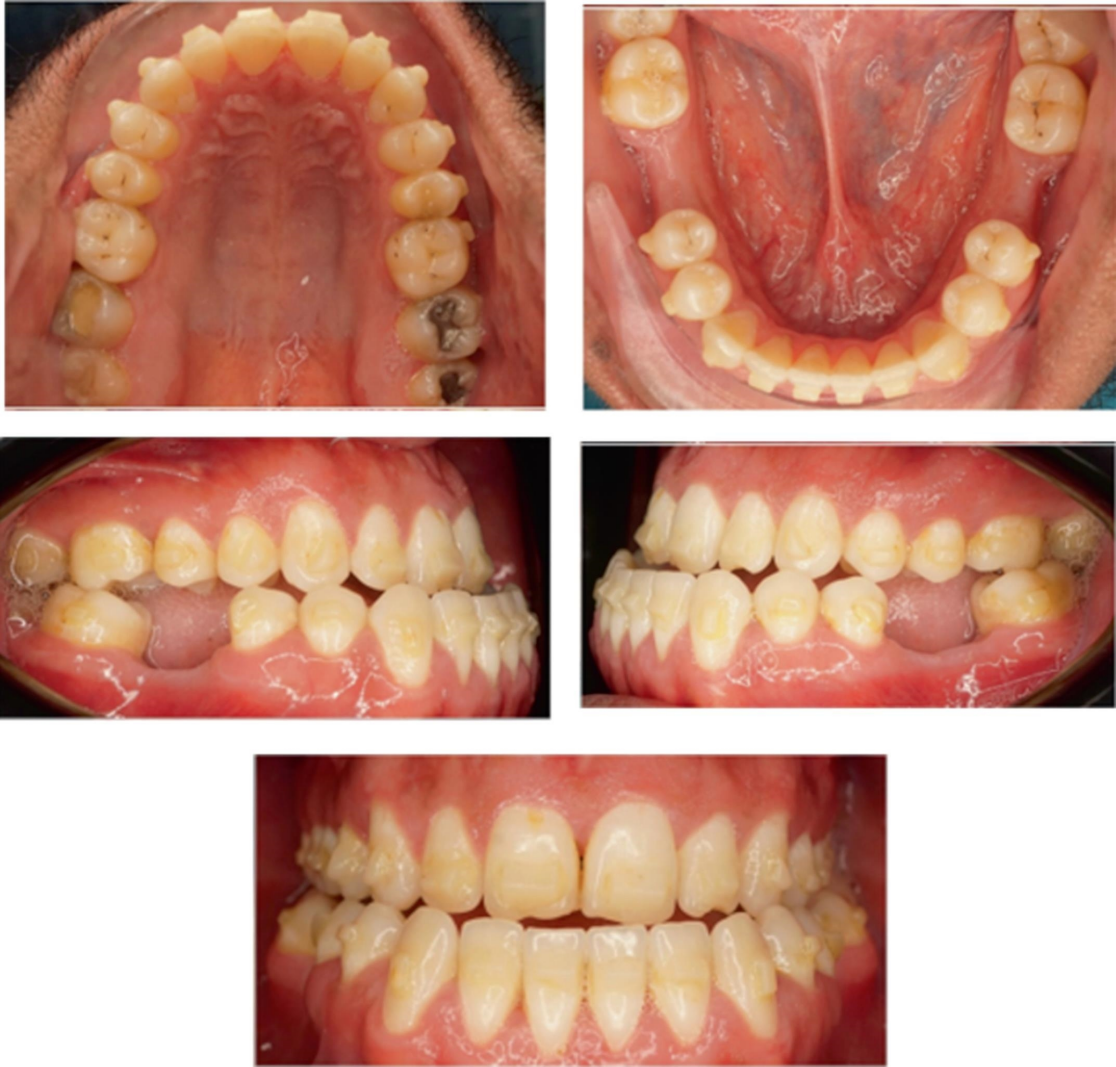
Presurgical intraoral photos: after aligners′ treatment.

**Figure 8:**
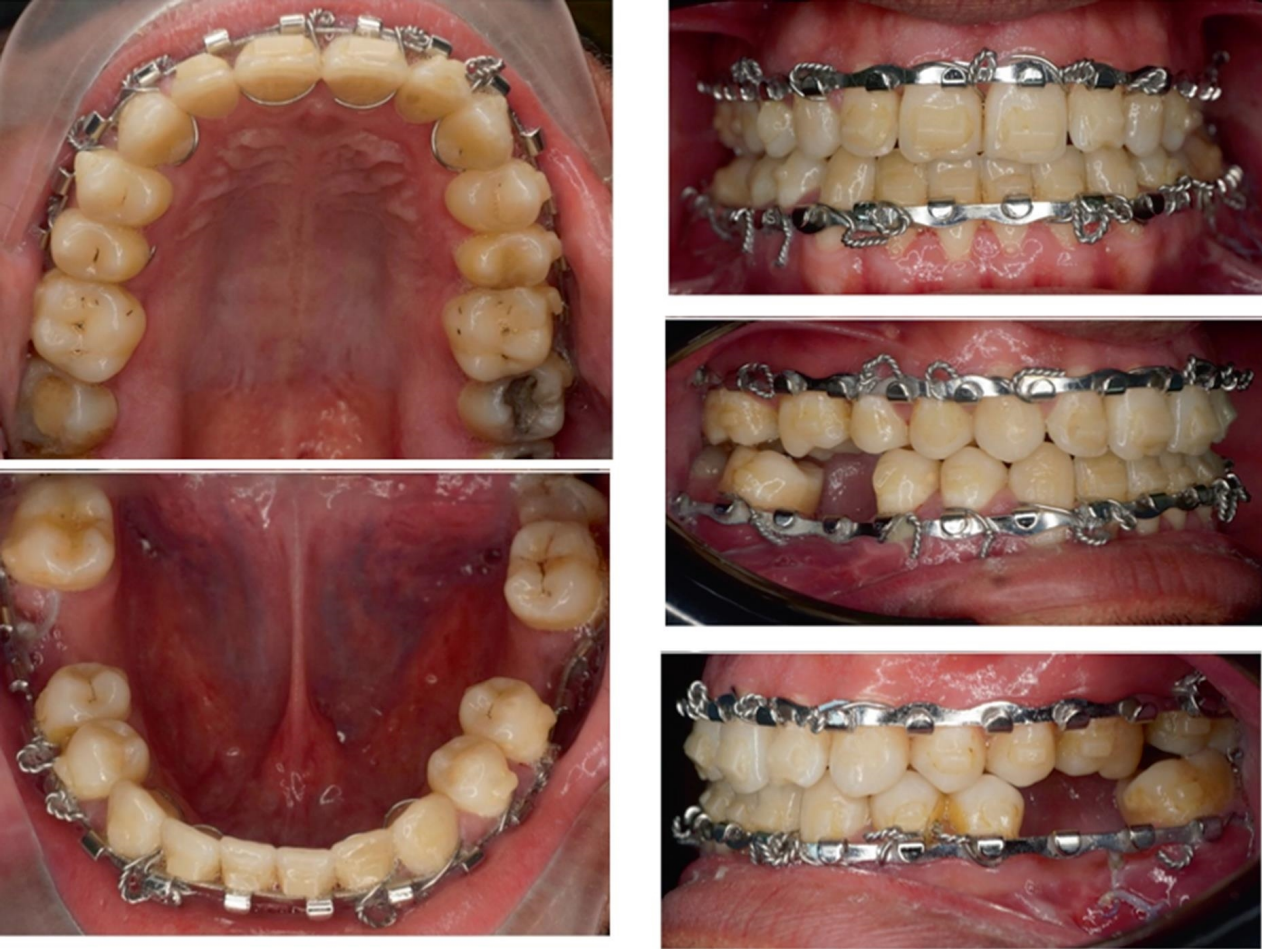
Postsurgical fixation using arch bar and elastics.

The treatment progress involved using clear aligners (Eon) along with an arch bar and orthognathic surgery. The aligners were used to decompensate the teeth and achieve a Class I canine relationship. The presurgical orthodontic biomechanics consisted of slight expansion of the maxillary arch and leveling and alignment of both the maxillary and mandibular arches using clear aligners. These mechanics were aimed at achieving appropriate dental decompensation prior to orthognathic surgery (Figures 6 and 7). The patient underwent mandibular setback surgery and postsurgical fixation using arch bar and elastics (Figure 8). The arch bar was removed, and the need for refinements or reusing the previous aligners was assessed. Attachments were removed, and retention was achieved using an upper Essix retainer and a lower fixed retainer from the canine to canine.

**Figure 9:**
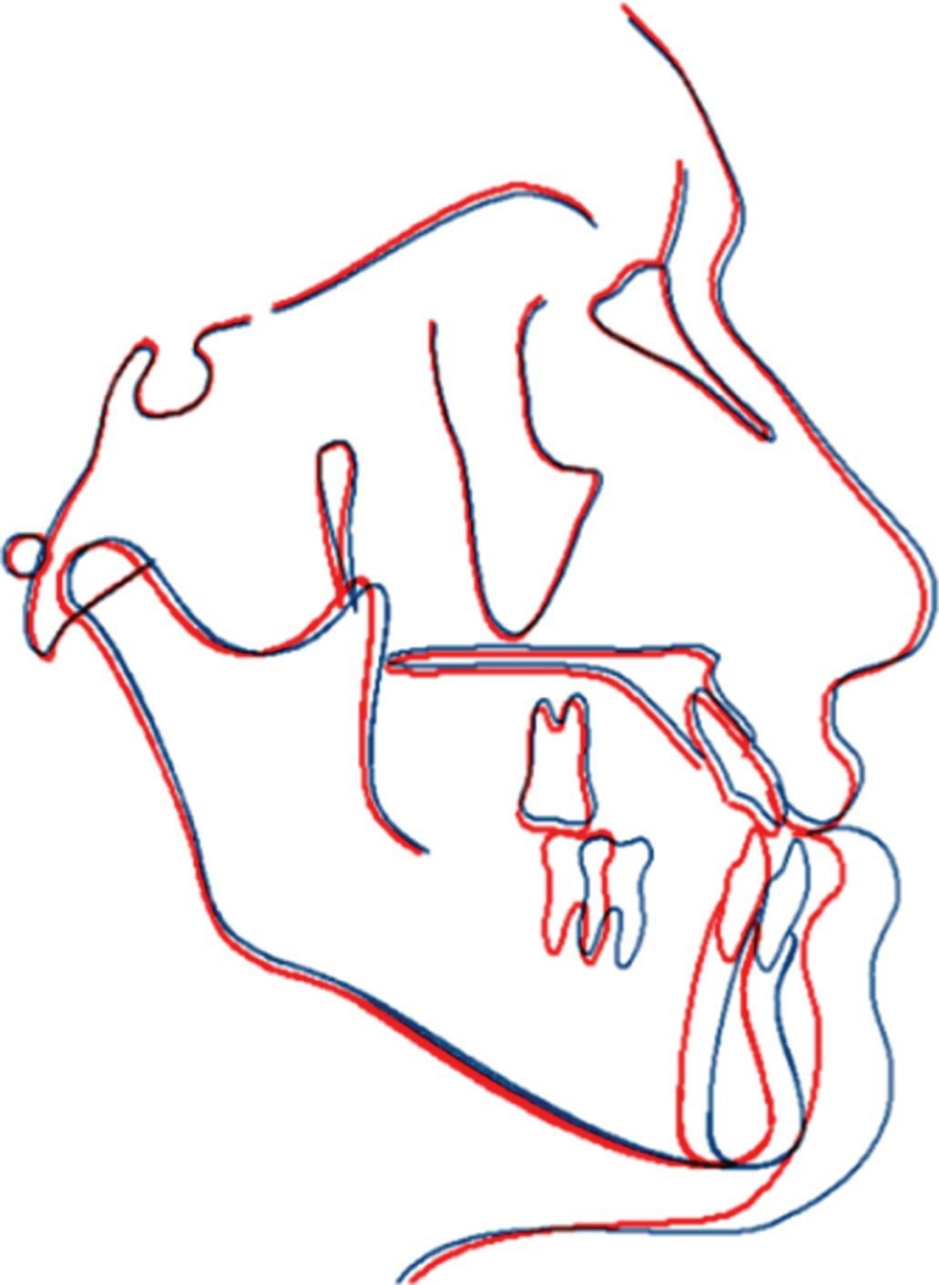
Superimposition. Presurgical, postsurgical: nose moved backward 1 mm. Upper lip moved downward 1 mm and backward 1 mm. Lower lip moved backward 6 mm. Soft tissue chin moved backward 6 mm. A point: the same. B point moved backward 5 mm. Pog point moved backward 5 mm.

Superimposition of the precephalometric and postcephalometric x‐rays was performed to assess the changes in the jaws, teeth, and soft tissue. This method aligned key anatomical landmarks for direct comparison and clearly showed the dental and skeletal modifications resulting from the orthognathic treatment (Figure 9 and Table 1).

**Table 1:** Cephalometric analysis numbers for pretreatment–presurgical–postsurgical.

Measurement	Norms	Pretreatment	Presurgical	Postsurgical
SNA	82^°^ ± 2^°^	80°	80°	80°
SNB	80^°^ ± 2^°^	86°	86°	80°
ANB	2^°^ ± 2^°^	−6°	−6°	0°
Wits	−1/0 ± 2 mm	−7 mm	−7 mm	−2 mm
SN‐Pog	80^°^ ± 3^°^	86°	86°	82°
NA‐A Pog	0^°^ ± 5.1^°^	−7°	−7°	−0.5°
SN‐PP	8^°^ ± 3^°^	10°	10°	10°
SN‐MP	32^°^ ± 5.1^°^	36°	36°	36°
PP‐MP	25^°^ ± 3^°^	29.3°	29.3°	29.3°
N‐S‐Gn	59.4^°^ ± 3.8^°^	63°	63°	63°
Me‐tgo‐Ar	126^°^ ± 10^°^	131°	131°	131°
ANS‐Me/N‐Me	55*%* ± 3*%*	59%	59%	59%
U1‐L1	131^°^ ± 5^°^	126°	133°	133°
U1‐SN	104^°^ ± 2^°^	115°	112°	112°
U1‐NA	22^°^ ± 5^°^ (4 ± 3 mm)	35°8 mm	31°7 mm	31°7 mm
L1‐NB	25^°^ ± 6^°^ (4 ± 2 mm)	28°8 mm	27°8.9 mm	27°5.4 mm
L1‐MP	93^°^ ± 6^°^	88°	87°	87°
Upper lip to E‐line	−4 ± 2 mm	−7 mm	−8 mm	−4 mm
Lower lip to E‐line	−2 ± 2 mm	−2 mm	−1 mm	−1 mm
Nasolabial angle	90°–110°	96°	99°	99°

**Figure 10:**
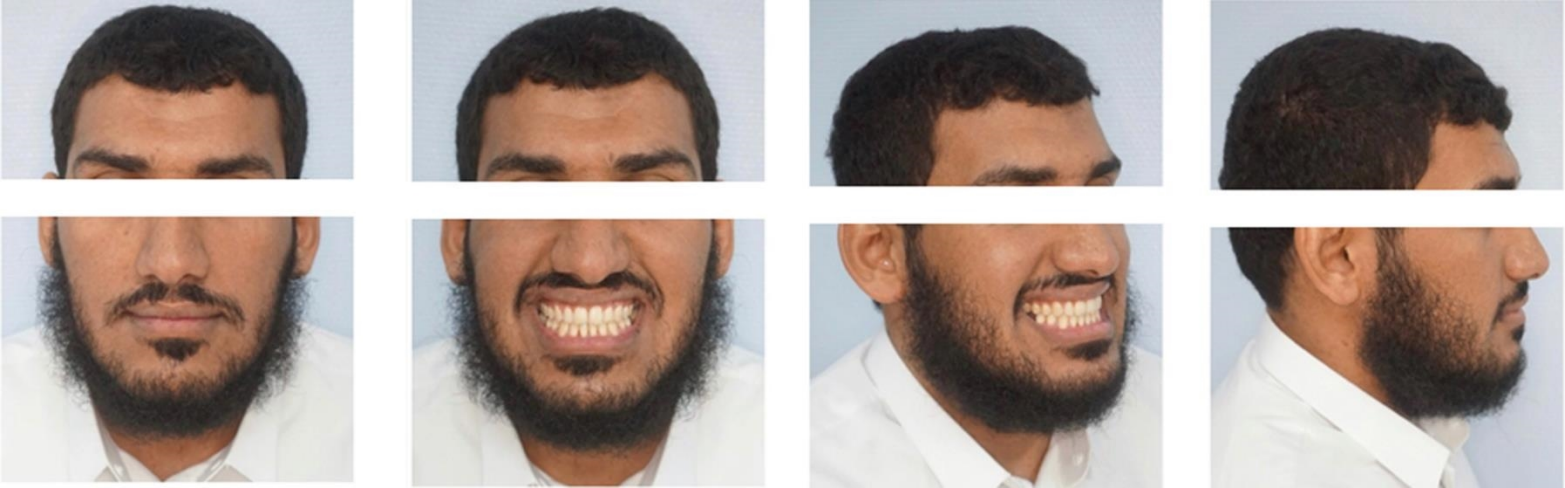
Final extraoral photos: Facial balance and profile were improved. Consonant smile arc was achieved.

**Figure 11:**
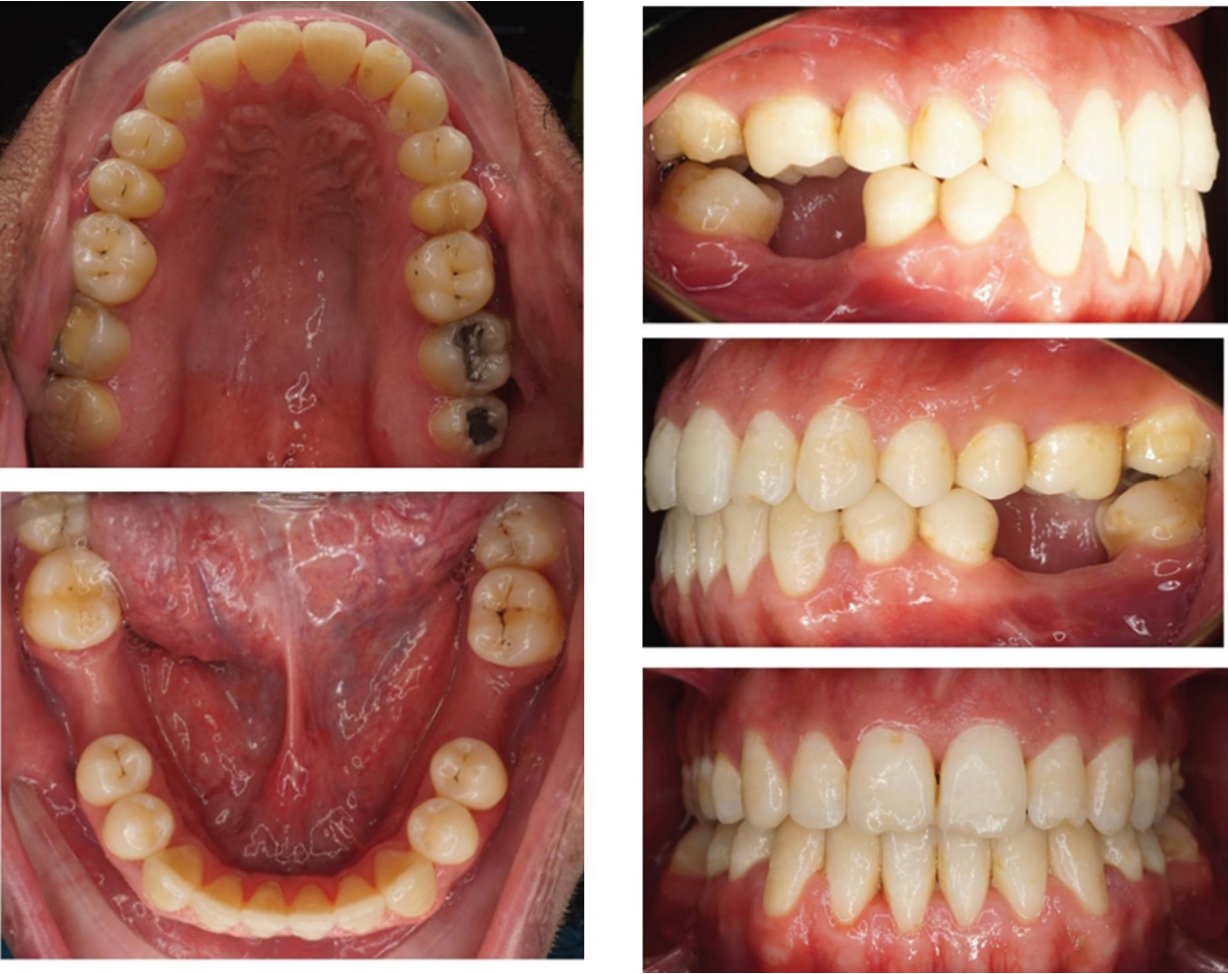
Final intraoral photos: A well interdigitation with Class I canine relationship on both sides was established. Positive overjet and an overbite were achieved. All teeth were aligned and leveled. Upper and lower incisors′ inclination was maintained. Lower midline shift was corrected.

**Figure 12:**
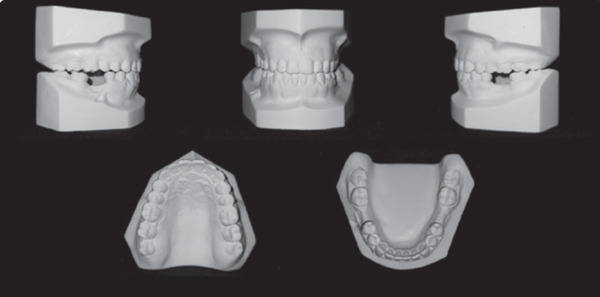
Final cast photos: Canine Class I relation right and left, the midline is on, overjet is 3 mm, overbite is 10%, Teeth #37 and 47 are mesially tilted, and Teeth #16 and 26 are slightly extruded.

**Figure 13:**
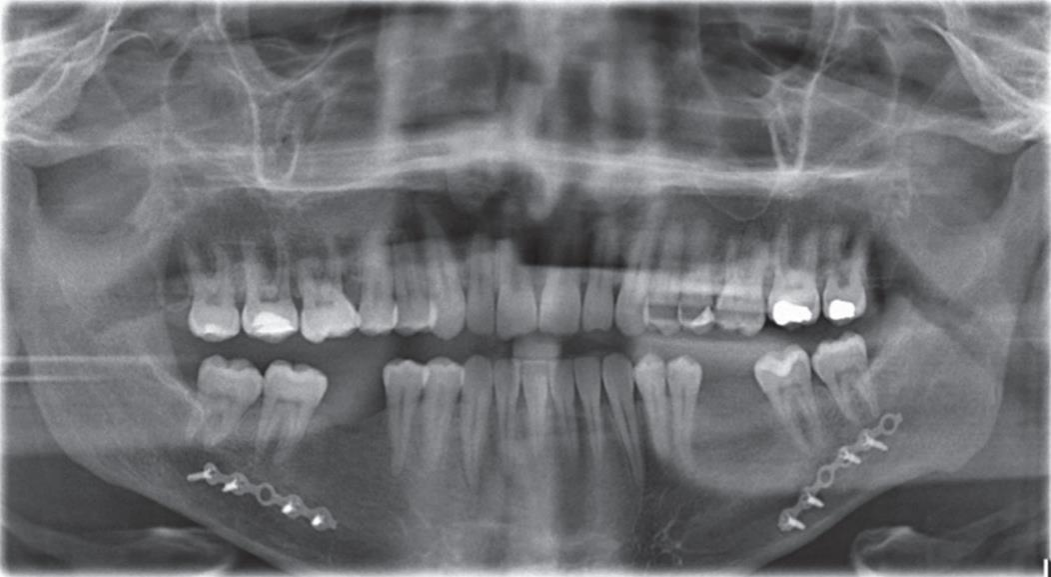
End of treatment panoramic view: Condyles maxillary sinus and bone trabeculation are within normal, fair root parallelism except #16, 23, and 34 (although accepted clinically).

**Figure 14:**
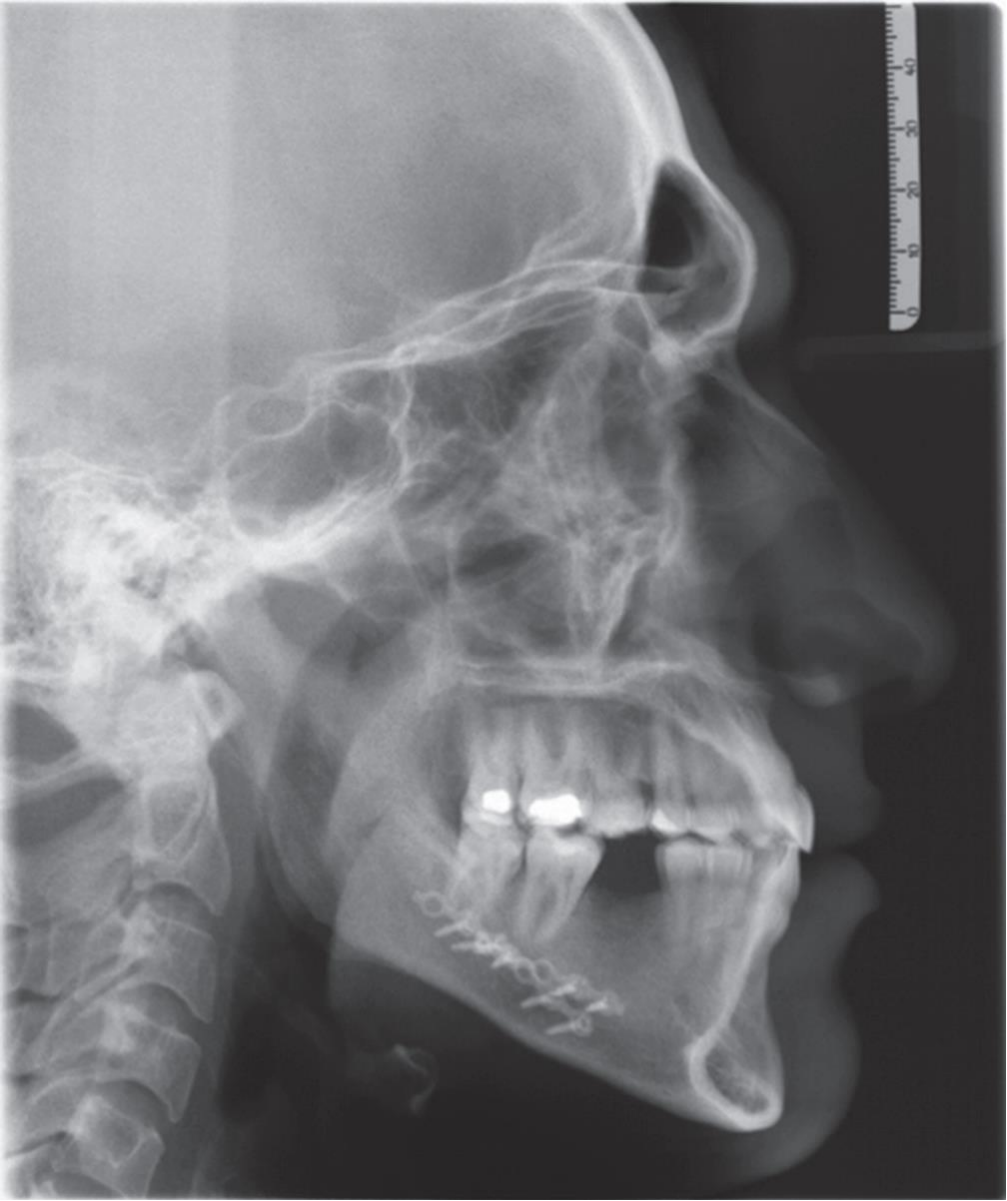
End of treatment lateral cephalometric radiograph: Class l skeletal pattern according to ANB and Wits, improved skeletal profile, average mandibular plane, normal proclination of upper and lower incisors, and average nasolabial angle.

The treatment outcomes showed improvement in the patient′s initial complaint, profile, and facial balance (Figure 10). A Class I canine relationship, positive overjet, and overbite were achieved. The lower midline shift was corrected, and a harmonious smile arc was achieved (Figures 11 and 12). Most of the roots showed fair parallelism, except for a few specific teeth (Figures 13 and 14).

## 3. Discussion

Angle′s Class III malocclusion is widely recognized as one of the most complex types of malocclusions to understand and treat optimally. It presents challenges in avoiding both overtreatment and undertreatment [15–17]. Orthognathic surgery is a procedure aimed at improving the relationship between the upper and lower jaws and enhancing facial profile. It is typically used for treating skeletal malocclusions that cannot be effectively addressed with conventional orthodontic treatment alone. Orthognathic surgery can address various abnormalities, including Class II and Class III skeletal angle malocclusions with anterior open bite and facial asymmetry, TMJ abnormalities, obstructive sleep apnea syndrome, post–cleft lip and palate, hemifacial microsomia, and deformities or malocclusions resulting from trauma [18].

Mandibular prognathism, categorized as a Class III malocclusion, occurs when the position of the mandible is more advanced than the cranial base. It is primarily caused by genetics, although environmental factors can also contribute. The “Habsburg jaw,” or mandibular prognathism, is a hereditary condition characterized by an underdeveloped mandible or a hypoplastic maxilla [19–21].

Orthognathic surgery is aimed at achieving facial and cranial structural balance by addressing maxillary discrepancies through osteotomy procedures within the masticatory system. During orthognathic surgery, the alignment of the maxilla and mandible is adjusted to establish proper dental posture and correct facial and maxillomandibular irregularities. The goal is to achieve a balanced relationship between the maxilla and mandible and align the teeth. The most commonly employed techniques in orthognathic surgery are sagittal split ramus osteotomy and intraoral vertical ramus osteotomy. Orthognathic surgery is frequently utilized to address conditions that impact chewing, facial pain, and aesthetics [22, 23].

The success of orthognathic surgery depends on achieving appropriate fixation and restoring normal occlusion. Temporary IMF with correct occlusal registration is necessary [24]. During the surgical correction of skeletal units, surgeons often rely on prefabricated surgical splints. These splints position the occlusion of opposing dental arches and stabilize the skeletal units at the new location. While rigid internal fixation with bone plates and screws provides long‐term stability and enables a quick return to function, the splint‐aided intraoperative IMF within the occlusal splint guides and determines the position for achieving rigid fixation. In select cases, a stabilizing splint may also be used in the postoperative phase to facilitate predictable healing [14].

In some cases, the maxilla and/or mandible may need to be divided into segments through osteotomy. During this surgical procedure, a patient‐specific arch bar is required to guide the eventual position of the jaw segments and enable postsurgical IMF [25]. Various methods have been used for IMF, including custom‐made arch bars, eyelet wires, and Schuchardt arch‐shaped splints made of metal and acrylic [26].

In the past, the most common method for achieving IMF involved placing arch bars on the maxillary and mandibular teeth using interdental wires. However, this method has disadvantages such as being time‐consuming, causing periodontal injury, causing pain to the patient during placement and removal, posing difficulties in maintaining oral hygiene, and presenting a significant risk of needle stick injury to the practitioner [24].

An alternative method for achieving IMF involves self‐tapping IMF screws, which have shown to be more effective compared to conventional Erich arch bars in treating mandibular fractures. Self‐tapping IMF screws offer advantages such as reduced operating time and a lower risk of needle stick injuries. Patients treated with IMF screws also tend to have better oral hygiene maintenance and acceptance compared to those treated with arch bars [27]. Conventional arch bars, followed by modified arch bars, provide more stability than IMF screws and may be preferred for patients requiring long‐term IMF [28].

Despite advancements in surgical planning and splint fabrication, achieving IMF and stabilizing the splint remain crucial for achieving predictable outcomes [14].

## 4. Conclusion

In orthodontic cases involving orthognathic surgery, the use of clear aligners treatment offers several advantages over conventional fixed orthodontic appliances. However, it is crucial to select an effective method of maxillomandibular fixation (IMF). This case report illustrates how a combination of clear aligners and a traditional arch bar splint as IMF during orthognathic surgery resulted in significant improvements in the patient′s facial profile and anterior crossbite. The treatment approach successfully met the patient′s demands and resulted in high levels of satisfaction with the treatment outcomes.

## Funding

No funding was received for this manuscript.

## Conflicts of Interest

The authors declare no conflicts of interest.

## Data Availability

The data that support the findings of this study are available from the corresponding author upon reasonable request.

## References

[1] Fleming P. S. , DiBiase A. T. , and Cobourne M. T. , The Aetiology of Malocclusion: A Contemporary View, Orthodontic Update. (2008) 1, no. 1, 16–21, 10.12968/ortu.2008.1.1.16.

[2] Alharbi F. , The Prevalence of Malocclusion Traits in Saudi Arabia 2015–2019: An Epidemiological Cross Sectional Study, Journal of International Oral Health. (2020) 12, no. 2, 129–134, 10.4103/jioh.jioh_200_19.

[3] Alogaibi Y. A. , Murshid Z. A. , Alsulimani F. F. , Linjawi A. I. , Almotairi M. , Alghamdi M. , Alharthy H. , and Hassan A. A. , Prevalence of Malocclusion and Orthodontic Treatment Needs Among Young Adults in Jeddah City, Journal of Orthodontic Science. (2020) 9, no. 1, 10.4103/jos.JOS_44_19. PMC7041309 32166082

[4] Albarakati S. F. and Taher S. , Malocclusion Traits in Saudi Females Seeking Orthodontic Treatment, Pakistan Oral & Dental Journal. (2010) 30, no. 1.

[5] Jamilian A. , Cannavale R. , Piancino M. G. , Eslami S. , and Perillo L. , Methodological Quality and Outcome of Systematic Reviews Reporting on Orthopaedic Treatment for Class III Malocclusion: Overview of Systematic Reviews, Journal of Orthodontics. (2016) 43, no. 2, 102–120, 10.1080/14653125.2016.1155334, 2-s2.0-84982238165. 27086590

[6] Rongo R. , D′Antò V. , Bucci R. , Polito I. , Martina R. , and Michelotti A. , Skeletal and Dental Effects of Class III Orthopaedic Treatment: A Systematic Review and Meta-Analysis, Journal of Oral Rehabilitation. (2017) 44, no. 7, 545–562, 10.1111/joor.12495, 2-s2.0-85017403487, 28214379. 28214379

[7] Galeotti A. , Martina S. , Viarani V. , Franchi L. , Rongo R. , D’Antò V. , and Festa P. , Cephalometric Effects of Pushing Splints 3 Compared With Rapid Maxillary Expansion and Facemask Therapy in Class III Malocclusion Children: A Randomized Controlled Trial, European Journal of Orthodontics. (2021) 43, no. 3, 274–282, 10.1093/ejo/cjaa076, 33313718. 33313718 PMC8186836

[8] Martina S. , Martina R. , Franchi L. , D′Anto V. , and Valletta R. , A New Appliance for Class III Treatment in Growing Patients: Pushing Splints 3, Case Reports in Dentistry. (2019) 2019, no. 1, 9597024, 10.1155/2019/9597024, 31827942. 31827942 PMC6885274

[9] Mulier D. , Gaitán Romero L. , Führer A. , Martin C. , Shujaat S. , Shaheen E. , Politis C. , and Jacobs R. , Long-Term Dental Stability After Orthognathic Surgery: A Systematic Review, European Journal of Orthodontics. (2021) 43, no. 1, 104–112, 10.1093/ejo/cjaa022, 32901268. 32901268

[10] Miyatake E. , Miyawaki S. , Morishige Y. , Nishiyama A. , Sasaki A. , and Takano-Yamamoto T. , Class III Malocclusion With Severe Facial Asymmetry, Unilateral Posterior Crossbite, and Temporomandibular Disorders, American Journal of Orthodontics and Dentofacial Orthopedics. (2003) 124, no. 4, 435–445, 10.1016/S0889-5406(03)00562-6, 2-s2.0-0141991100, 14560275. 14560275

[11] Dastgir R. , Bemudez P. F. , Valiathan M. , Baur D. A. , and Quereshy F. A. , The Use of Clear Aligners in Orthognathic Surgeries: A Case Series, Oral Surgery, Oral Medicine, Oral Pathology and Oral Radiology. (2024) 137, no. 3, e22–e40, 10.1016/j.oooo.2023.07.016, 38160198. 38160198

[12] Caminiti M. and Lou T. , Clear Aligner Orthognathic Splints, Journal of Oral and Maxillofacial Surgery. (2019) 77, no. 5, 1071.e1–1071.e8, 10.1016/j.joms.2018.12.012, 2-s2.0-85061376945. 30664865

[13] Aziz S. R. , Clear Aligner Orthognathic Surgery: An Overview, Frontiers of Oral and Maxillofacial Medicine. (2022) 4, 10.21037/fomm-21-18.

[14] Taub D. I. and Palermo V. , Orthognathic Surgery for the Invisalign Patient, Seminars in Orthodontics. (2017) 23, no. 1, 99–102, 10.1053/j.sodo.2016.10.008, 2-s2.0-85008457438.

[15] Stellzig-Eisenhauer A. , Lux C. J. , and Schuster G. , Treatment Decision in Adult Patients With Class III Malocclusion: Orthodontic Therapy or Orthognathic Surgery?, American Journal of Orthodontics and Dentofacial Orthopedics. (2002) 122, no. 1, 27–37, 10.1067/mod.2002.123632, 2-s2.0-15944400979, 12142894. 12142894

[16] Leonardi R. , Muraglie S. , Lo Giudice A. , Aboulazm K. S. , and Nucera R. , Evaluation of Mandibular Symmetry and Morphology in Adult Patients With Unilateral Posterior Crossbite: A CBCT Study Using a Surface-to-Surface Matching Technique, European Journal of Orthodontics. (2020) 42, no. 6, 650–657, 10.1093/ejo/cjz106, 31995170. 31995170

[17] Lo Giudice A. , Rustico L. , Caprioglio A. , Migliorati M. , and Nucera R. , Evaluation of Condylar Cortical Bone Thickness in Patient Groups With Different Vertical Facial Dimensions Using Cone-Beam Computed Tomography, Odontology. (2020) 108, no. 4, 669–675, 10.1007/s10266-020-00510-2. 32236830

[18] Larsen M. K. , Indications for Orthognathic Surgery-A Review, OHDM. (2017) 16, no. 2, 1–13.

[19] Doraczynska-Kowalik A. , Nelke K. H. , Pawlak W. , Sasiadek M. M. , and Gerber H. , Genetic Factors Involved in Mandibular Prognathism, Journal of Craniofacial Surgery. (2017) 28, no. 5, e422–e431, 10.1097/SCS.0000000000003627, 2-s2.0-85020201592, 28570402. 28570402

[20] Sun R. , Wang Y. , Jin M. , Chen L. , Cao Y. , and Chen F. , Identification and Functional Studies of MYO1H for Mandibular Prognathism, Journal of Dental Research. (2018) 97, no. 13, 1501–1509, 10.1177/0022034518784936, 2-s2.0-85049824461, 29986156. 29986156

[21] Kantaputra P. N. , Pruksametanan A. , Phondee N. , Hutsadaloi A. , Intachai W. , Kawasaki K. , Ohazama A. , Ngamphiw C. , Tongsima S. , Ketudat Cairns J. R. , and Tripuwabhrut P. , ADAMTSL1and Mandibular Prognathism, Clinical Genetics. (2019) 95, no. 4, 507–515, 10.1111/cge.13519, 2-s2.0-85063095551. 30714143

[22] Sousa C. S. and Turrini R. N. , Complications in Orthognathic Surgery: A Comprehensive Review, Journal of Oral and Maxillofacial Surgery, Medicine, and Pathology. (2012) 24, no. 2, 67–74, 10.1016/j.ajoms.2012.01.014, 2-s2.0-84870545211.

[23] He P. , Iwanaga J. , Matsushita Y. , Adeeb N. , Topale N. , Tubbs R. S. , and Kusukawa J. , A Comparative Review of Mandibular Orthognathic Surgeries With a Focus on Intraoral Vertico-Sagittal Ramus Osteotomy, Cureus. (2017) 9, no. 12, 10.7759/cureus.1924. PMC7394221 32760640

[24] Rothe T. M. , Kumar P. , Shah N. , Shah R. , Mahajan A. , and Kumar A. , Comparative Evaluation of Efficacy of Conventional Arch Bar, Intermaxillary Fixation Screws, and Modified Arch Bar for Intermaxillary Fixation, Journal of Maxillofacial and Oral Surgery. (2019) 18, no. 3, 412–418, 10.1007/s12663-018-1110-7, 2-s2.0-85070433498. 31371884 PMC6639526

[25] Song Y. L. , Yu N. , Danny B. P. , and Chew M. T. , A Pilot Study on Three-Dimensional Printing of Stainless Steel Arch Bars for Orthognathic Segmental Jaw Surgeries, Annals of 3D Printed Medicine. (2022) 6, no. 6, 100055, 10.1016/j.stlm.2022.100055.

[26] Schuchardt G. J. , Ein Beitrag zur chirurgischen Kieferorthpadie unter Berucksichtigung ihrer fur di Behandlung angeborener und erworbener Kiefer deformitaten bei soldaten, Dtsch Zahn Mund Kieferheilkd. (1942) 9, 73–89.

[27] Nandini G. D. , Balakrishna R. , and Rao J. , Self Tapping Screws V/S Erich Arch Bar for Inter Maxillary Fixation: A Comparative Clinical Study in the Treatment of Mandibular Fractures, Journal of Maxillofacial and Oral Surgery. (2011) 10, no. 2, 127–131, 10.1007/s12663-011-0191-3, 2-s2.0-84863963227, 22654363. 22654363 PMC3177518

[28] Schneider A. M. , David L. R. , DeFranzo A. J. , Marks M. W. , Molnar J. A. , and Argenta L. C. , Use of Specialized Bone Screws for Intermaxillary Fixation, Annals of Plastic Surgery. (2000) 44, no. 2, 154–157, 10.1097/00000637-200044020-00005, 2-s2.0-0033951239, 10696041. 10696041

